# Outline of an evolutionary morphology generator towards the modular design of a biohybrid catheter

**DOI:** 10.3389/frobt.2024.1337722

**Published:** 2024-04-12

**Authors:** Michail-Antisthenis Tsompanas, Igor Balaz

**Affiliations:** ^1^ School of Computing and Creative Technologies, University of the West of England, Bristol, United Kingdom; ^2^ Unconventional Computing Laboratory, University of the West of England, Bristol, United Kingdom; ^3^ Laboratory for Meteorology, Physics and Biophysics, Faculty of Agriculture, University of Novi Sad, Novi Sad, Serbia

**Keywords:** biohybrid machines, 3D voxel-based simulator, optimization, evolutionary algorithms, machine learning

## Abstract

Biohybrid machines (BHMs) are an amalgam of actuators composed of living cells with synthetic materials. They are engineered in order to improve autonomy, adaptability and energy efficiency beyond what conventional robots can offer. However, designing these machines is no trivial task for humans, provided the field’s short history and, thus, the limited experience and expertise on designing and controlling similar entities, such as soft robots. To unveil the advantages of BHMs, we propose to overcome the hindrances of their design process by developing a modular modeling and simulation framework for the digital design of BHMs that incorporates Artificial Intelligence powered algorithms. Here, we present the initial workings of the first module in an exemplar framework, namely, an evolutionary morphology generator. As proof-of-principle for this project, we use the scenario of developing a biohybrid catheter as a medical device capable of arriving to hard-to-reach regions of the human body to release drugs. We study the automatically generated morphology of actuators that will enable the functionality of that catheter. The primary results presented here enforced the update of the methodology used, in order to better depict the problem under study, while also provided insights for the future versions of the software module.

## 1 Introduction

Although well-established robotic theory have produced great tools for humanity in a macroscale setting, the same principles are not trivially transferable to machines that require to be built in millimeter scales or lower. This challenge stems from the inherent complexities associated with manufacturing at such miniature scales, where machines built within these dimensions often fail to match the performance of their larger-scale counterparts (Rus and Tolley, 2015). When comparing conventional rigid robots with unconventional soft robots that can also be driven by biomaterial, what can be defined as biohybrid machines (BHMs), the latter have several advantages in microscale settings. For instance, BHMs can better mimic the movement pattern and the structure of living systems, they have increased power density and lifetime span in smaller scales, while they have the potential to self-heal, assembly and recreate depending on the application (Lin et al., 2022). However, as BHMs are composed of living cells and synthetic materials, their designing process is not straightforward and not yet validated like their conventional counterparts.

Recognizing that the existing body of work predominantly emphasizes single demonstrators and keeping in mind the imperative of scalability for BHMs, the design process requires meticulous attention to manufacturing and assembly procedures (Menciassi et al., 2020). Consequently, we advocate for a modular software framework where rudimentary designs will be automatically created and selected using AI algorithms, followed by gradual refinement through more precise simulations of the evolving complex system. While a similar approach was explored previously (Kriegman et al., 2020), our work specifically addresses constraints connected with the design of biohybrid catheters as medical devices. One of the most important issues is to take into account the biocompatibility of materials used in catheter construction. Hence, through Poisson’s ratio specification, we constrain our BHMs to biocompatible soft materials, such as silicones (e.g., PDMS, Dragonskin, and Ecoflex) or thermoplastic polymers (e.g., polyurethane or Pemba) commonly used for fabricating commercial devices. Additionally, anticipating minimal mechanical resistance due to the use of biological actuators in the biohybrid catheter, we set the Young’s modulus at 5 Mpa.

At this stage of development we are primarily interested in mechanical material properties. However, since catheter is an invasive device, special attention should be taken about material biocompatibility, chemical stability, low thrombogenicity, ease of sterilization and compatibility with medications it will come in contact with during medical procedures. All of these aspects will constitute additional constraints on the choice of materials in the final product development, and will require more refined simulation modules.

Establishing such AI-powered BHM design process is one of the primary goals of our ongoing project, BioMeld^1^. In this project, we further aim to harness this framework to pioneer the development of a groundbreaking medical device–a biohybrid catheter designed to deliver medications to hard-to-reach regions within the human body. With this specific medical device application in mind, we defined initial testing scenarios to better represent the problem at hand. It is important to note that the adaptability of our framework extends beyond this context; it can be applied to diverse applications with slight adjustments to assumptions, constraints, and testing scenarios.

Given that the first design and testing steps will be performed purely *in silico*, a physics simulator is needed. Voxelyze is a fully open source physics simulator that is written in platform agnostic C++ (Hiller and Lipson, 2014). While there is a large body of work on physics simulators used for designing robotics, the majority of widely established simulators are studying rigid-body interactions (Hwangbo et al., 2019). On the other hand, there are simulators that can study soft material, thus soft robotics, but they mainly adopt small deformations of homogeneous materials that have external forces applied to them (Nealen et al., 2006). As a result, Voxelyze is preferred as it manages to model quantitatively and computationally efficiently the non-linear deformation of heterogeneous soft bodies, along with the statics and dynamics in a 3D environment.

Evolutionary methodologies are employed to discover possible morphologies as it was proved that they can overpower human designers’ capacity. An important meta-parameter in evolutionary methodologies is the representation of possible solutions (Ryerkerk et al., 2017; Tsompanas et al., 2021). Its choice mainly depends on the problem, while the level of advantages may vary. Here, given the unique nature of the problem we want to tackle, we chose Compositional Pattern Producing Networks (CPPNs) (Stanley, 2007). Specifically, the main objective of the morphology generator for our case is to create solutions that enable the catheter to achieve efficient angular movement. Previous research has demonstrated that such efficient movement is more attainable when repeating patterns of passive and active tissues are present within an organism (Cheney et al., 2014). As a result, the use of CPPNs is an ideal option for the application that we study.

The methodology presented here is the first step within a comprehensive framework that will enable the automated design of complicated BHMs for diverse applications. These applications will be determined based on the information and constraints provided as inputs to the design process. In this context, we present and analyze the preliminary results to elucidate the revisions and enhancements made to the software framework, aligning it more closely with the specific problem under consideration. The insights garnered from these results inform our ongoing efforts to implement the BHMs design process, as we continue to explore and assess various strategies.

## 2 Background

The framework of Voxelyze can provide a truly heterogeneous material simulation (Hiller and Lipson, 2014), as it is capable of assuming objects that can be composed of any number of material types with different physical properties (i.e., density, stiffness, Poisson’s ratio and friction coefficients). This attribute can accommodate the recent technological advances in multi-material additive manufacturing and bio-engineering (Ricotti et al., 2017). On top of external forces that can be applied in Voxelyze simulated objects, a mechanism of volumetric actuation is included in the simulation framework that allows the investigation of innovative arrangements, such as BHMs. The framework is built in a computationally efficient way so that standard desktop computers can handle simulation of bodies with multiple degrees of freedom.

Soft robots or BHMs that are designed with the use of CPPNs representation and evolutionary methods (Cheney et al., 2014; 2015; Kriegman et al., 2020) tend to contain patterns of same material that resemble the morphology of tissues in natural bodies (i.e., a group of actuating voxels similar to a muscle attached to a group of passive voxels similar to a bone). Consequently, these pattern-composed morphologies are more efficient when the objective is movement, since the group of voxels located in adjacent locations can enhance their ability to achieve locomotion in a coordinated manner. On the contrary, when direct encoding is used, the initial random deposition of different materials next to each other ensues in each voxel working autonomously and sometimes in an antagonistic manner to its adjacent voxels. As a result, there is no coordination or emergence of the similar behavior and the characteristics of voxels’ action remain local. Moreover, the champions of evolutionary rounds are similar to the randomly initialized ones, and their fitness improvement is minuscule (Cheney et al., 2014).

There are several types of indirect representations and an interesting category of them is that of developmental encoding. This class draws inspiration from developmental biology and is set to simulate the mapping of a genotype to the appropriate phenotype via a procedure resembling growth. CPPNs that are used here, are an intriguing example of this class, as they can express the structural relation of voxels that would be the product of a developmental process. Namely, their formalism is similar to that of artificial neural networks (ANNs), with the difference of using a variety of activation functions and differentiated topologies. The topology is unconstrained and can represent any possible relationship.

**FIGURE 1:**
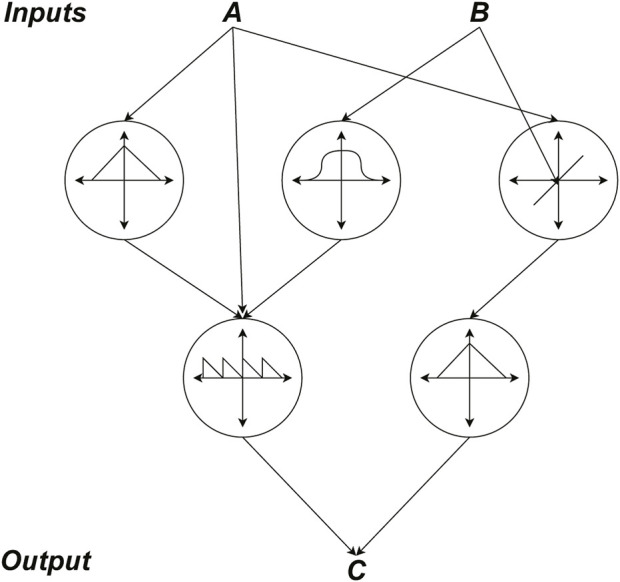
A paradigm of CPPN architecture that has two inputs **(A,B)** and one output **(C)**. Each node in the hidden layer represents a specific function and weighted connections represent the total input of each node.

Taking into account that CPPNs have a similar formalism as ANNs (as depicted in Figure 1), we can readily apply efficient methodologies and strategies proposed for ANNs onto CPPNs and expect comparable results (Stanley, 2007). Thus, the evolutionary optimization method Neuroevolution of Augmenting Topologies (NEAT) can be tested on CPPNs to evolve progressively more complex networks with promising efficiency (Stanley and Miikkulainen, 2002; Stanley et al., 2009). However, the application of AFPO (Age-Fitness-Pareto-Optimization) (Schmidt and Lipson, 2010) has achieved the evolution of increasingly complex phenotypic expression morphologies in previous works (Kriegman et al., 2020). These morphologies exhibit regularities and symmetries that are fine-tuned through the track of generations, thus, AFPO will be used here as the optimization procedure.

The inspiration for this work comes from the framework published by Kriegman et al. (2020), where software tools are used to generate morphologies that are then synthesized *in vitro* and compared with the simulation results. That work studies fitness functions for four applications. However, all of these applications are related to the maximum displacement of the organism or other objects that are manipulated by the organism. Also, the evolved morphologies are standalone machines that are restricted only to match a predefined size. In our work, additional constraints are required to represent the more complicated behaviour since the produced morphologies will be parts of a more complex machine (the BHM catheter). Regarding the fitness function, our goal is to ensure maximal angular displacement, and we explore various approaches to determine the optimal way of defining the desired behavior. Initially, the implementations focused on utilizing maximum displacement over three dimensions as a fitness function. However, as this approach revealed certain limitations, we further investigated displacement over a two-dimensional plane.

## 3 Methods

The first step of a broader BHM modular modeling and simulation framework is the evolutionary morphology generator presented here that provides an initial morphology of the BHM to be tested and optimized in different scenarios. While the rest of the modeling and simulation framework will be more accurate and targeting more complex parts of the BHM, the initial morphology is generated from scratch without relying on extensive prior knowledge of the problem’s specific details. Instead, it is based on a high-level description of what defines a desirable solution, namely, a fitness function (i.e., achieving angular movement), and a set of constraints associated with the problem (i.e., one end of the morphology being fixed). In our case, the fitness function can be the angle that the BHM catheter actuator can bend by itself. Consequently, the proposed first module utilizes a genetic algorithm optimization methodology to invent a fit solution for this roughly defined problem.

The genetic algorithm is employed to initiate a population of possible morphologies, test them against a fitness function and choose the fittest individuals to produce more and novel morphologies to be tested. As the main objective of the morphology generator for our case is to create solutions that enable the catheter to achieve efficient angular movement, the use of CPPNs as an encoding mechanism of individuals within the genetic algorithm is an ideal option for the application that we study.

Several BHM morphologies are created and tested against the fitness function in the soft-body physics engine Voxelyze on a 3D Cartesian lattice workspace, where voxels (the basic building block in Voxelyze) are connected together to form a functional machine. The design is set so that at each voxel located in a unique *x*, *y* and *z* coordinate, can be characterized as one of three basic types: 1) passive, 2) actuated with fixed physical properties (i.e., density, stiffness, Poisson’s ratio and friction coefficients), and 3) absent - to indicate that in that area no material will be used. Also, environmental parameters, like gravitational acceleration and friction to voxels in contact with the surface plane, are selected to reflect an initially predefined testing scenario. The parameters used here are illustrated in Table 1 and are in accordance with previous works (Kriegman et al., 2020). Despite the initially chosen material parameters and the number of different materials, this does not limit the generalization ability of the system, namely, choosing multiple different materials with a variety of density, stiffness, Poisson’s ratio and friction coefficient values. Moreover, Voxelyze can represent morphologies with great spatial complexity, following recent technological advances in multi-material additive manufacturing and bio-engineering (Ricotti et al., 2017). Thus, limitations that were present in other manufacturing processes do not need to be incorporated in the proposed designing method.

**TABLE 1:** Parameters of active and passive voxel.

Parameters	Active voxel	Passive voxel
Elastic modulus (*MPa*)	5	5
Density (*kg*/*m* ^3^)	1,000	1,000
Poisson’s ratio	0.35	0.35
Coefficient of Thermal Expansion (1/°*C*)	0.01	0
Coefficient of static friction	1	1
Coefficient of dynamic friction	0.5	0.5
Frequency of actuation (*Hz*)	2	N/A

The implementation studied here uses the Cartesian coordinates in a 3D space as input nodes of the CPPN, while the output nodes provide the voxel type of the simulated area of the BHM to be designed (passive, contractile or absent). Also, each node in the hidden layer represents a mathematical function, while connections between nodes represent function compositions. The connections are weighted in such a way that the output of a function is multiplied by the weight of its outgoing connection. If multiple connections feed into the same function, it means that the downstream function takes the sum of their weighted outputs. The specific parameters used for CPPNs in this study are provided in Table 2.

**TABLE 2:** Key parameters of CPPN architectures.

Parameter	Values
Input nodes	5 (*x*, *y*, *z* that are Cartesian coordinates of voxel *d* the distance from the centre and 1 is the bias)
Output nodes	2 ([Empty space or not] and [active or passive voxel])
Hidden layer nodes	Initial value in the range of [4,10]
Possible equations	sine, absolute value, negative absolute value
used in hidden	square, negative square, square root
layer nodes	and negative square root
Connection weights range	[-1,1]

Consequently, the genotype is a CPPN, while the phenotype is the morphology of a BHM actuator, produced by utilizing that CPPN, using voxel coordinates as inputs, while the output of the network is the functionality of the appropriate voxel. The application of all possible combinations of coordinates as inputs, will result in the definition of an output pattern of the given structure morphology in the workspace. The geometry of a BHM is encoded by a bit-string. It is noteworthy here that CPPN encoding is a scale-free mapping, so BHMs of any desired resolution can be designed by the same encoding without further optimization rounds.

For simplicity and in order to follow the successful implementation presented and tested previously (Kriegman et al., 2020), in the first version of this module of the design pipeline, we implemented a fundamental evolutionary optimization algorithm similar to AFPO. The next versions of the software will include more sophisticated evolutionary methodologies (such as NEAT (Stanley et al., 2009)) that will be tailored for better performance.

Here, the optimization methodology follows the procedure described in (Kriegman et al., 2020): 50 randomly generated CPPN-encoded BHM morphologies are initially produced. An additional 50 individuals are injected into the population that are the result of mutations of the initial 50 individuals. One more individual is randomly produced, so the population reaches 101 individuals. Each one of them is decoded into a BHM morphology that is tested with the Voxelyze software (if not already evaluated) and evaluation experiments begin for each BHM after a given time (1 s of simulated time) for it to settle under gravity. The evaluation of each morphology lasts 10 s of simulated time. The fitness function is defined in the following section for each reported set of experiments, and it can be the total displacement of the center of mass of the BHM in general or on the *YZ* plane. After the evaluation of all the individuals in the population, the 50 best performers are returned to the evolution loop. There is no crossover operator in this version of the software for simplicity purposes, however, future versions will test the crossover contribution to the evolution.

The meta-parameters of the algorithm are selected to follow the initial implementation of the code, however, they can be optimized for applications in future versions. Specifically, the population size is selected to be 50 individuals. The termination criterion is set to be 2000 generations being evaluated, whereas the computational budget is selected to be 48 h of wall-time (unless otherwise mentioned). When either is reached, the experimental run will terminate. The mutation operator is applied to all the individuals with probability of 1/6 for each of the six kinds of mutations (add, modify, or remove a vertex and add, modify, or remove an edge). Thus, both the architecture and the weights of the network can be altered per mutation operation. Nonetheless, if the product of a mutation is neutral (namely, no difference in the morphology produced from the alteration in CPPNs), an additional mutation operation is performed until 1500 unsuccessful tries are tested.

After the methodology reaches the computational budget, the results were analysed manually and appropriate changes in the methodology or the constraints were applied. In that way, we aimed to better depict the problem under study in the virtual environment which provides the initial morphologies for the actuators of BHM catheters that will be used in the next steps of our planned framework.

## 4 Results

In this section the preliminary results of the module will be presented. Even though the methodology is still in an inceptive phase, the following analysis triggered the update of the methodology and effectively enabled the next versions of the module to better reflect the problem at hand. For all the sets of experiments the parameters of the voxels that constitute the BHM designs are provided in Table 1 and maintained the same throughout all the experiments presented in this Section. Also, all experiments run on a system with Intel Xeon CPU E5-2650 with 48 cores at 2.20 GHz and 64 GB RAM.

### 4.1 First set of experiments

**FIGURE 2:**
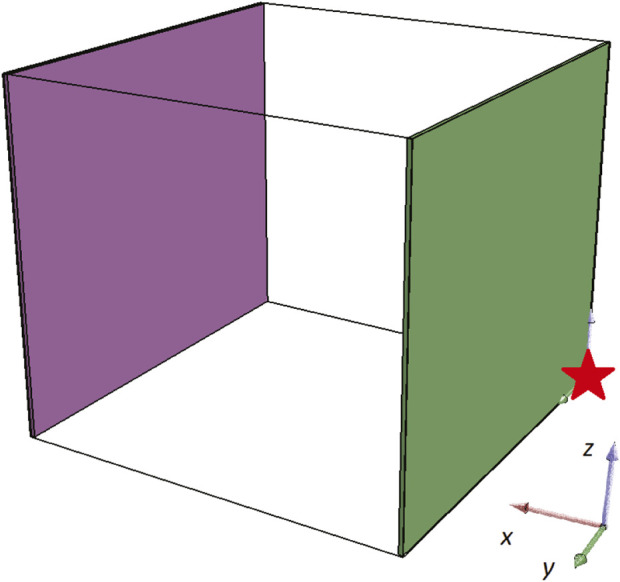
Boundary conditions set in the simulation environment for all individuals.

In the first set of the experiments, the functionality of the framework was tested in order to be adapted to the problem at hand (i.e., designing a BHM catheter actuator), by altering only the boundary conditions of the available design area within the simulation. The boundary conditions are set as illustrated in Figure 2. Note that the red star indicates the (0,0,0) coordinates. In detail, the green plane is a fixed boundary condition for translation and rotation displacement of all the voxels that are adjacent to that plane (i.e., plane *YZ* for *X* = 0). Moreover, the purple plane (i.e., plane *YZ* for *X* = 8) is set to a free boundary condition, meaning that all the adjacent voxels to that plane are free to move in whatever way the simulation dictates. The rest of the setting is maintained the same as with the original open-source code. Most importantly, the available design area is set to 8 × 8 × 7 voxels and the fitness function is defined as the maximum displacement of the center of mass of the designed BHM after 10 s of simulated time (given one more second for the morphology to settle under gravity at the start of the simulation). Consequently, the basic description of the problem here is defined as a 8 × 8 × 7 configuration of voxels that can be active, passive or empty (with the aforementioned physical characteristics) and the whole configuration should be fixed on one end, to imitate the entry point of a catheter, whereas the distal catheter tip is free to move and navigate into a tortuous network of vessels.

**FIGURE 3:**
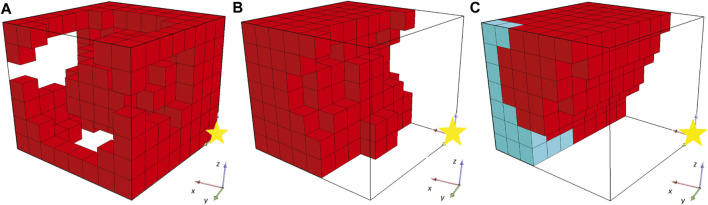
Champions of generations **(A)** 0, **(B)** 5 and **(C)** 1976 for the first set of experiments.

**FIGURE 4:**
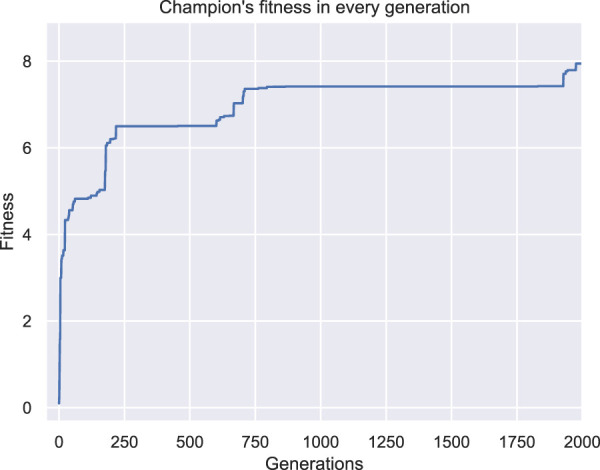
Fitness of the champion of each generation throughout evolution for the first set of experiments.

While the optimization methodology starts with some interesting individuals (i.e., the ones that are randomly generated, Figure 3A) and the fitness values are constantly increasing (as illustrated in Figure 4), the champion after five generations (Figure 3B) has a unique characteristic. As illustrated in Figure 3B (where the yellow star indicates the point with coordinates (0,0,0)), the produced morphology is not attached to the fixed plane. This means that during evolution, the morphologies manage to “escape” the definition of the problem. Thus, this solution/individual better suits the description of a BHM that moves the furthest of the initial position rather than perform a bending movement. The rest of individuals produced through evolution, follow the same concept (i.e., the final champion after 1976 generations that is depicted in Figure 3C). This situation demonstrates perfectly that evolutionary methodologies tend to take advantage of weak points in the description of the problem, the constraints of the problem, or simulators that introduce levels of abstraction to effectively reflect real phenomena in digital computers. This is also apparent from the graph of the fitness of the champion in every generation that reaches the value 8 at the end of the 2000 generations (Figure 4). The fitness here is the displacement of the center of mass of the morphology in voxel lengths.

**FIGURE 5:**
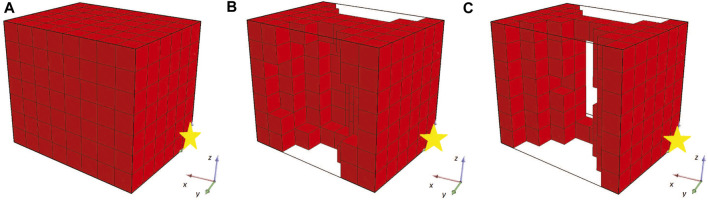
Champions of generations **(A)** 0, **(B)** 7 and **(C)** 71 for the first set of experiments after a new constraint was introduced.

**FIGURE 6:**
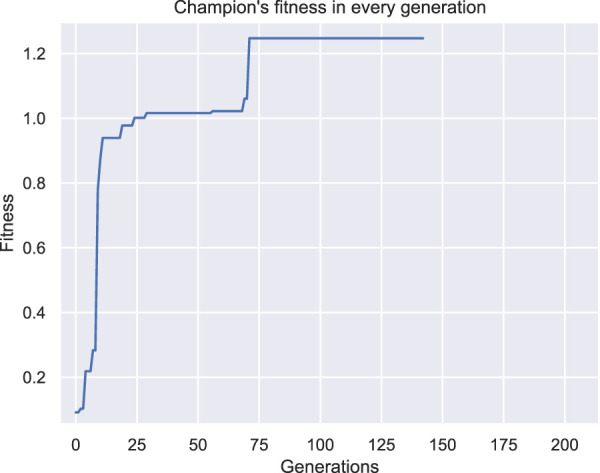
Fitness of the champion of each generation throughout evolution for the first set of experiments after a new constraint was introduced.

**FIGURE 7:**
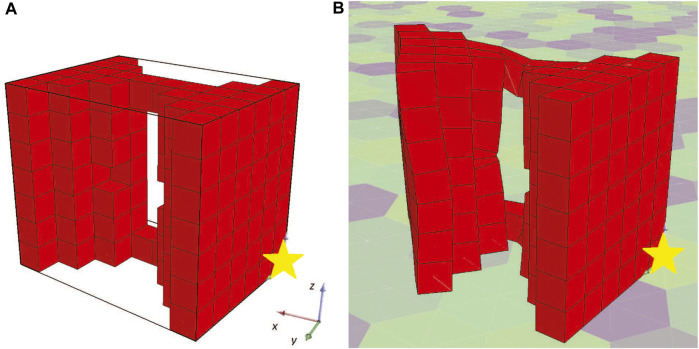
Champion of the first set of experiments after a new constraint was introduced **(A)** at the starting point and **(B)** after 10 s of simulated time.

As a result, this setting was not duplicated. On the contrary, to better describe the problem, an additional constraint was inserted into the methodology. In specific, the morphology should be attached to the plane *YZ* with *X* = 0, otherwise the morphology design is considered invalid and another individual is produced through the mutation operator. The results after the new constraint was included are shown in Figure 5. Also, the fitness of the champions throughout evolution is illustrated in Figure 6. Here, the individuals only consist of muscle tissue (i.e., active voxels) and as dictated by the new constraint all of them are attached to the plane *YZ* at *X* = 0. Even though no other champion is found after generation 71 that can outperform the current one (as depicted in Figure 6), an interesting individual is discovered after the evaluation of the predefined amount of generations. In Figure 7 the initial state of the actuator is depicted alongside the final state after the 10 s of simulated time. It can be observed that the actuator bends so that its free end is now at a location around 60° from the initial position. Moreover, although the fitness of the second run individuals (Figure 6) is significantly lower than the ones discovered in the first run (Figure 4), it is obvious that the ones discovered during the second run fit better the objective, despite the smaller fitness values. This is because of the insufficiently defined constraints.

### 4.2 Second set of experiments

Taking into consideration the results from the first set of experiments and aiming to achieve a BHM morphology that can bend at even larger angles, the maximum *X* dimension was increased from 8 to 20 voxels. In that way the produced morphologies would better depict a BHM actuator that can bend the catheter’s body to greater angles. Consequently, all the parameters were kept fixed, except for the available voxels in *X* dimension, resulting in a slightly updated basic description of the problem; namely, updating only the last description’s dimensions to 20 × 8 × 7 voxels. Note that this alternation significantly decelerated the simulations and, as a result, the following experiments would not reach the 2000 generations stopping criterion, but the computational budget limit of 48 h.

**FIGURE 8:**
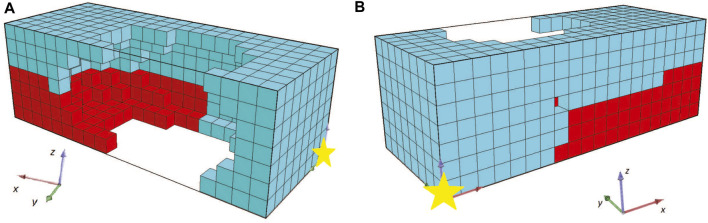
Champion of the first run for the second set of experiments from two **(A,B)** perspectives.

**FIGURE 9:**
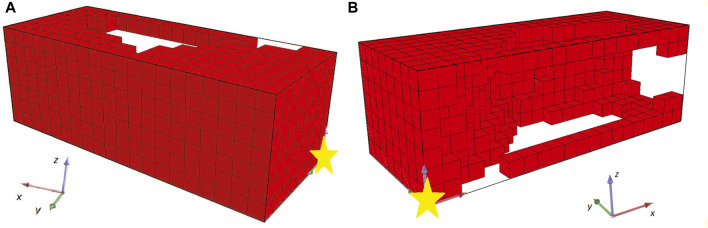
Champion of the second run for the second set of experiments from two **(A,B)** perspectives.

**FIGURE 10:**
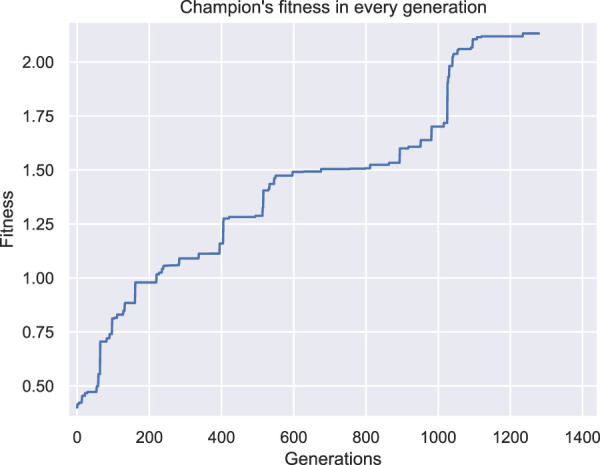
Fitness of the champion of each generation throughout evolution for the second set of experiments first run.

**FIGURE 11:**
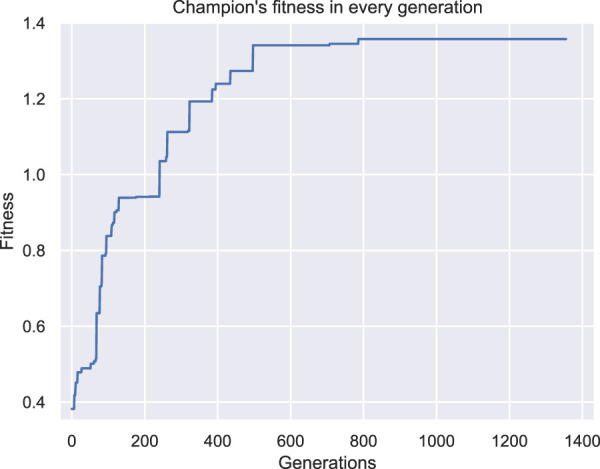
Fitness of the champion of each generation throughout evolution for the second set of experiments second run.

In Figures 8, 9 the champions of this configuration are illustrated for two independent runs with different initial populations. Note that two perspectives of each individual are given in each figure and the voxel with coordinates (0,0,0) is illustrated with a yellow star in all the figures as a point of reference. Both champions are the best individuals found after 48 h of wall time execution of the optimization algorithm (the one in Figure 8 was originally discovered at generation number 1235, while the one in Figure 9 at generation number 786, as depicted in Figures 10, 11 respectively).

**FIGURE 12:**
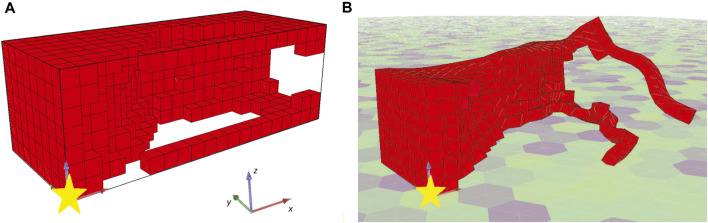
**(A)** Initial and **(B)** final position (after 10 s of simulated time) of the second run champion morphology.

Both individuals are intriguing morphologies for a BHM actuator, however, the desired behavior of greater angles compared with the results from the first set of experiments is not achieved here. On the contrary, evolutionary optimization seems to have pinpointed again a weak area in the definition of the problem and exploited it by producing individuals that obtain high fitness values without fitting the problem’s specification. In fact, in Figure 12 the initial position of the second champion and its position after the 10 s of simulated movement are illustrated. The morphology has two features that can be characterized as tentacles that are separated from the main body of the morphology and, thus, can oscillate away from the main body. The calculation of the center of mass total displacement takes into account all the voxels’ mass and location. As a result, the development of tentacles manages to distort the fitness function. While the simulated actuator does not bend or turn significantly from the initial position (when compared with previous results), the tentacles end up notably away from their initial position. This means that the center of mass has significantly moved away from the initial position, thus, higher values in the fitness function have been achieved, without the desired functionality being present in the currently evolved morphologies. This can be observed by the fitness values achieved throughout evolution generations by the champions in each run in Figures 10, 11.

### 4.3 Third set of experiments

To tackle this faulty behavior, we included the following alternation in the description of the problem. The fitness function was updated to better reflect the objective of the evolved BHMs and the desired behavior that they need to have. Thus, instead of measuring the total displacement of the center of mass for the morphology, now the fitness function is defined as the displacement of the center of mass in the *YZ* plane. This is calculated in terms of the Euclidean distance that the projection of center of mass on the *YZ* plane from its starting point to the point it rests after 10 s of simulated time. As a result, a clearer goal is set for the evolution, whereas the manipulation of the previous fitness function by morphologies with oscillating tentacles and movement on the *X*-axis is now discouraged.

Additionally, to better manifest the desired morphologies’ design space and given that the BHM actuator needs to be within a flexible bioreactor chamber, one more change was included. Specifically, all the possible actuator morphologies were enclosed in a frame of soft material or consisting of voxels with parameters as defined in Table 1 for the passive type of voxels. Note that all the other parameters are the same as the previous set of experiments. Thus, in line with the newly introduced fitness function, the problem description is now revised to generate an 18 × 6 × 5 configuration of voxels surrounded by a passive voxel enclosure with a thickness of one.

**FIGURE 13:**
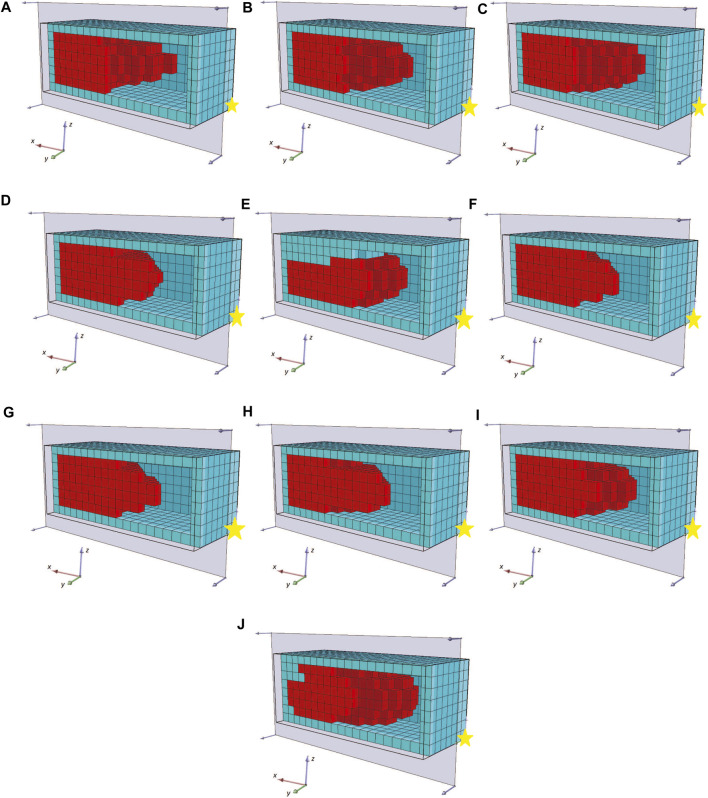
The champions of the 10 independent runs **(A–J)** of evolutionary optimization for the third set of experiments in a front section view.

**FIGURE 14:**
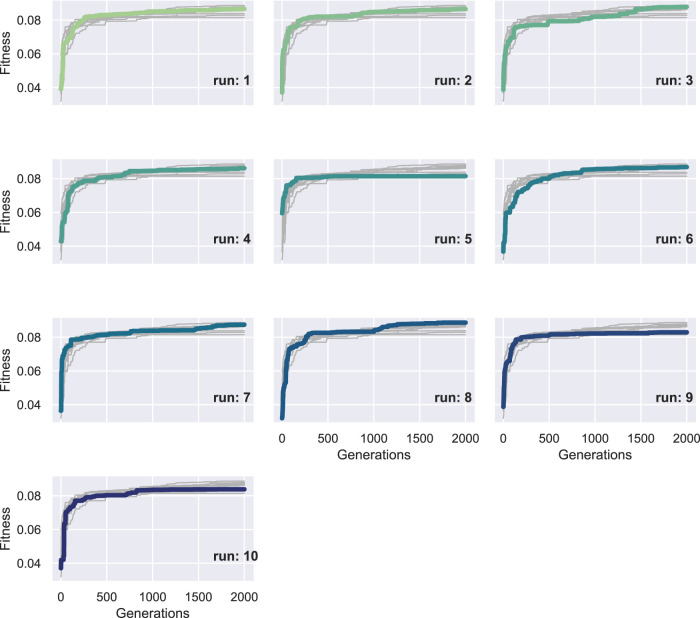
Fitness of the champion of each generation throughout evolution for each of the ten runs in the third set of experiments. The bold lines represent the fitness of the current run, while the thin lines the fitness of the rest of runs for comparison measure.

**FIGURE 15:**
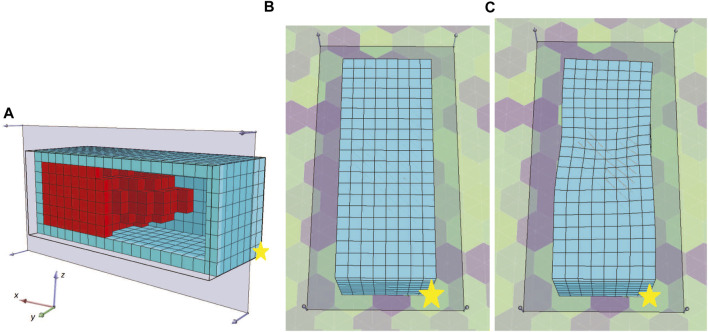
**(A)** Front section view **(B)** initial and **(C)** final position of the first champion (Figure 13) for the third set of experiments.

To better depict the morphologies, the front section view (at the *XZ* plane for *Y* = 8) of the champions of the 10 independent runs of this set of experiments are shown in Figure 13. For reference, the voxel with coordinates (0,0,0) is marked with a yellow star. We performed 10 independent runs to verify the results. The addition of the soft material frame around the evolved BHMs provided an additional obstacle to the morphologies’ ability to bend or turn. Thus, all the champions presented in Figure 13 manage to move only slightly on each way of the *X*-axis, as a result of the oscillations of the active voxels being accumulated to the free end of the morphology. The lower achieved fitness values (or the displacement of the center of mass in the *YZ* plane) can also be distinguished in Figure 14, where the maximum value at the end of the 2000 generations only slightly overcomes the 0.08 of a voxel length. There is a notable decrease in fitness values and movement (compared with the results from the first and second sets). In order to demonstrate the behavior of the morphologies evolved during these experiments, the initial and final position of the first champion (Figure 13A) is illustrated in Figure 15.

## 5 Discussion

This work demonstrates a proposed first stage of a modular modeling and simulation framework for the digital design of BHMs. The benchmark for the optimization is a physics simulator named Voxelyze which is a fully open-source physics simulator that is written in platform agnostic C++. Voxelyze can provide a truly heterogeneous material simulation and can simulate a mechanism of volumetric actuation that enables the investigation of BHMs.

While some previous research has attempted to design robotic morphologies using evolutionary methods, relying on rigid elements from a predefined set has imposed serious constraints on the complexity of the outcomes. In contrast, by drawing inspiration from nature, we can observe that organisms consist of a diverse array of cells, including both rigid (e.g., bones) and soft (e.g., muscles) types, arranged in groups of varying shapes. Nature’s ability to stack these heterogeneous materials to form novel morphologies has facilitated the evolution of highly complex and efficiently mobile organisms.

The use of direct encodings in the evolutionary optimization process restricts the search space and makes it challenging for regularities in morphologies to naturally emerge, which are essential for coordinated behaviors. On the other hand, employing indirect encodings, such as CPPNs, allows for the evolution of highly complex morphologies with regular and symmetrical patterns, with or without variations, as highlighted in previous work (Stanley, 2007). Furthermore, it is worth noting that CPPNs’ tendency towards symmetry and regularity simplifies *in vitro* production of BHMs (Kriegman et al., 2020).

The goal of the methodology presented here is to generate an initial morphology without relying on detailed prior knowledge of the problem. Instead, it leverages a basic description of what qualifies as a desirable solution. The simulator’s parameters have been configured to model two distinct types of materials, each possessing fixed physical properties (density, stiffness, Poisson’s ratio, and friction coefficients). One of these materials is passive, while the other is responsible for actuating to facilitate the necessary movement of the BHM actuator. Additionally, the simulation allows for the incorporation of empty spaces within the BHM morphology, following the previously published methodology (Kriegman et al., 2020). This choice was made to allow for the emergence of more complex configurations, potentially leading to more elaborate movement patterns. While empty space was a concern in prior studies (Kriegman et al., 2020) due to the healing ability of living cells to fill small empty spaces, we have opted not to include a build-filter in this work. This decision is based on the absence of small empty spaces in any of the configurations identified in the final set of experiments (depicted in Figure 13).

As the objective of the BHM is efficient angular movement and based on the fact that movement can be achieved more efficiently when repeating motifs of passive and active tissues are present, the use of CPPNs is suitable to act as a representation scheme for the individuals of the evolutionary optimization algorithm. As CPPNs have a similar formalism as ANNs, we can readily apply efficient methodologies and strategies proposed for ANNs. A good candidate is NEAT methodology that evolves progressively more complex networks. However, for the first version of the software module, AFPO methodology was implemented for simplicity reasons. The evolutionary process is repeated until the computation budget is reached and the best-performing individual in terms of the fitness function (i.e., the champion of the evolutionary run) is the output of this first module of the pipeline.

Through experimentation with the code and after the analysis of the outputs, updates of the methodology and the constraints were applied to more accurately mimic the problem under study. Given the predominant focus on individual examples in this field, and with the objective of enhancing the scalability of the BHMs design process, in this paper we demonstrated the adaptation of the code to the specific medical device problem and recommend implementing this methodology as the initial phase within a software framework featuring various levels of abstractions. The overarching goal of this framework is to advance the fabrication of BHMs toward a more applicable pipeline.

Starting with insufficient constraints, the produced morphologies evade the problem specifications, despite providing large fitness values. Thus, additional constraints were added. When we studied just a cuboid actuator we realised that it can bent only at lower acute angles. The next version of the code allowed for longer dimensions to enable larger bending angles. However, the problematic definition of the fitness function kept outputting invalid solutions. Consequently, the fitness function was updated and the methodology was confined with one more constraint, namely, the enclosure of the actuator into an elastic frame. The latest results do not perform as well as the ones in the first set of experiments, because of the increased resistance of the aforementioned frame, however, future work aspects can tackle this problem. Namely, we plan to implement more material types in the methodology and use more sophisticated evolutionary algorithms.

The presented methods represent the initial phase of a software framework with progressive levels of abstraction, aiming to shift the manufacturing of BHMs towards a bio-intelligent paradigm and model-based engineering. Our plan involves developing a self-monitoring and self-controlling manufacturing pipeline for BHMs. To realize such a pipeline, we need to streamline design, quoting, manufacturing, verification, and reporting processes, thus significantly reducing error-prone manual steps. Given that BHMs involve living cell actuators, which greatly expand the parameter space, we propose leveraging AI-guided modeling to optimize the search for the most efficient design. The subsequent steps include experimental testing, optimization, and verification through the creation of a proof-of-principle re-configurable modular catheter. Ultimately, we aim to consolidate all necessary manufacturing equipment into an integrated bio-intelligent manufacturing cell, demonstrating its adaptable operation.

Aiming towards a self-monitoring and self-controlling manufacturing pipeline, the simulation-optimization framework will require to eventually become independent from supervision of human experts. Nonetheless, the results presented here have characteristically demonstrated the capacity of evolutionary optimization methodologies to exploit weak points in problem definitions or get deceived into local optima (if a robust definition of the problem is considered). Thus, the current methodology, similar to previous works (Kriegman et al., 2020), includes an expert-in-the-loop that have to update the fitness functions, constrains of the problem or the simulator settings to closer approximate *in vitro* results. To alleviate this limitation, our future work will focus around more robust definitions of the problem by automatically analysing data and behaviours of *in vitro* prototypes to tackle the possible reality-gap (Salvato et al., 2021). On the other hand, the deception of EAs by specific areas of the fitness landscape has been previously studied and there are different approaches that proved to be able to address that (i.e., novelty search optimization (Lehman and Stanley, 2011; Tsompanas et al., 2020)). Similar techniques can be included in future iterations of the proposed methodology.

## Data Availability

The raw data supporting the conclusion of this article will be made available by the authors, without undue reservation.

## Footnotes

1
https://biomeld.eu/
